# Choroid plexus and perivascular space abnormalities in CerTra syndrome: neuroimaging and histological findings

**DOI:** 10.3389/fneur.2026.1792598

**Published:** 2026-04-13

**Authors:** Chenyang Li, Dominique Leitner, Huize Pang, Laura Gould, Orrin Devinsky, Christopher William, Thomas Wisniewski, Youssef Zaim Wadghiri, Jiangyang Zhang, Yulin Ge

**Affiliations:** 1 Department of Radiology, Bernard and Irene Schwartz Center for Biomedical Imaging, New York University Grossman School of Medicine, New York, NY, United States; 2 Comprehensive Epilepsy Center, NYU Grossman School of Medicine, New York, NY, United States; 3 Department of Neurology, NYU Grossman School of Medicine, New York, NY, United States; 4 Center for Cognitive Neurology, NYU Grossman School of Medicine, New York, NY, United States; 5 Sudden Unexplained Death in Childhood Foundation, Roseland, NJ, United States; 6 Department of Pathology, NYU Grossman School of Medicine, New York, NY, United States; 7 Department of Psychiatry, NYU Grossman School of Medicine, New York, NY, United States

**Keywords:** case report, *CERT1* variant, ChP cyst, pediatric neuropathology, perivascular space, SUDC

## Abstract

Ceramide transporter syndrome (CerTra syndrome) is a rare neurodevelopmental disorder caused by pathogenic variants in *CERT1* gene encoding ceramide transporter (CERT). These variants disrupt ceramide transport and sphingolipid homeostasis, leading to a clinical phenotype that includes developmental delay, movement abnormalities, and structural brain anomalies. Despite growing recognition of this condition, detailed neuroimaging and neuropathological characterization remain limited. Here, we present a 12-year-old girl with a pathogenic *CERT1* variant complicated by sudden unexplained death, who presented with unreported neuroimaging abnormalities in choroid plexus (ChP) and perivascular space (PVS). Clinical Magnetic Resonance Imaging (MRI) obtained approximately 10 years prior to death, as well as postmortem MRI, revealed bilateral cystic enlargement of the ChP and prominent PVS filled with abundant proteinaceous material in white matter. Neuropathological examination demonstrated marked ChP epithelial disorganization, reduced aquaporin-1 (AQP1) expression, cyst formation, and focal calcifications, which may be associated with disturbances in cerebrospinal fluid (CSF) dynamics. These findings raise the possibility that *CERT1* variants may be associated with ChP architectural changes and altered perivascular clearance.

## 1 Introduction

Ceramide Transporter Syndrome (CerTra syndrome, OMIM #604677) is a rare neurodevelopmental disorder associated with pathogenic variants in *CERT1* (1–4), the gene encoding the ceramide transporter (CERT). CERT plays a critical role in sphingolipid metabolism by mediating the transfer of ceramide for sphingomyelin synthesis. Several disease-associated *CERT1* variants disrupt the autoregulatory phosphorylation of CERT, resulting in constitutive activation of the transporter and increased sphingolipid synthesis, consistent with a gain-of-function mechanism (5–8). Affected individuals exhibit varying degrees of infantile hypotonia, global developmental delay, motor delay, and intellectual disability (3). Additional clinical features may include speech delay, behavioral abnormalities, high pain tolerance, feeding difficulties, and seizures. Due to the limited number of reported cases and the rarity of CerTra syndrome, the neuroimaging features associated with this condition remain insufficiently characterized, and definitive conclusions have yet to be established. In a recent study by Gehin et al. (3), neuroimaging findings commonly reported in CerTra syndrome include a thin corpus callosum, ventriculomegaly, delayed myelination, and cerebellar atrophy. In CerTra syndrome, most pathogenic variants arise *de novo* and are heterozygous. These variants cluster within regulatory domains essential for CERT phosphorylation and inactivation. Collectively, this evidence supports the current notion that *CERT1* variants produce a consistent neurodevelopmental phenotype linked to dysregulated sphingolipid metabolism.

The ChP plays a central role in cerebrospinal fluid (CSF) production and regulation of the brain’s CSF homeostasis (9), while PVS are key components of the glymphatic system, facilitating CSF-interstitial fluid exchange and metabolic waste clearance (10). Disruption of glymphatic function has been implicated in multiple neurodevelopmental (11–15) and neurodegenerative disorders (16–18). However, its involvement in *CERT1*-related pathology has not been explored. *CERT1* variants may disrupt cellular integrity, barrier function, and lipid-dependent membrane properties, though downstream neuropathological consequences are not well understood. To our knowledge, ChP abnormalities and PVS alterations have not previously been described in individuals with *CERT1* variants.

In this report, we describe a rare pediatric case involving a 12-year-old individual with a pathogenic *CERT1* variant complicated by sudden unexplained death in childhood (SUDC). Antemortem MRI, postmortem MRI, and neuropathological analyses were conducted to systematically correlate *in vivo* imaging findings with *ex vivo* and histological observations. Multimodal evaluation identified previously unreported abnormalities of the ChP and PVS, delineating a novel neuroimaging phenotype that may underline the observed clinical features.

## 2 Materials and methods

### 2.1 Clinical and brain specimen information

The patient was a 12-year-old female with a heterozygous *de novo CERT1* variant and clinically consistent with CerTra syndrome. She was born in full term via vaginal delivery, complicated by postnatal oxygen desaturations requiring neonatal intensive care unit (NICU) support. Her developmental course was notable for global delay affecting both cognitive and motor domains. She was non-verbal and had chronic sleep disturbance. Additional medical history included left-sided dystonia and asthma. The patient experienced a sudden, unexpected sleep-related death at 12 years of age, no epilepsy or other neurological disorder has been diagnosed for this case. Additionally, no family history of neurodevelopmental disorders or sudden unexplained death was reported. Furthermore, based on the available autopsy records, gross cardiac inspection, including the coronary arteries, revealed no structural abnormalities and no diagnostic findings were identified for cause of death. However, no cardiac histopathological findings were reported.

The whole brain tissue of this patient was obtained through the multisite collaboration with the Sudden Unexplained Death in Childhood Registry and Research Collaborative (SUDCRRC), approved by the NYU Grossman School of Medicine Institutional Review Board. SUDCRRC evaluates cases ages 1 month to 18 years old who died suddenly and unexpectedly, and the cause of death is unexplained after autopsy and complete forensic assessment (19). Consent for SUDC cases was provided by the decedent’s parent(s)/guardian(s). This case report was conducted in accordance with institutional guidelines and reported following the Care Report (CARE) guidelines.

### 2.2 Genetic evaluation

Clinical genetic evaluation was performed in 2024 after the patient’s death. Trio exome sequencing was conducted in the proband and both biological parents to investigate potential monogenic causes contributing to the patient’s neurodevelopmental phenotype and sudden death. Sequencing was performed using paired-end 150-bp chemistry on the Illumina NovaSeq platform. Library preparation utilized the IDT xGen Exome Research Panel V1.0, with alignment to the GRCh37/hg19 reference genome. Bioinformatic annotation and variant prioritization were performed using the Ambry Variant Analyzer (AVA) pipeline. Variants were filtered based on inheritance model, population frequency, predicted functional impact, and clinical relevance. Relevant findings underwent confirmation either by automated fluorescence dideoxy sequencing or by evaluation of coverage and alternate read ratios exceeding established confidence thresholds, with manual review by molecular geneticists using Integrative Genomics Viewer (IGV). Variant interpretation and classification were performed in accordance with American College of Medical Genetics and Genomics (ACMG) guidelines. Mitochondrial genome analysis was not performed as part of this assay.

### 2.3 Antemortem clinical MRI

Antemortem brain MRI was performed as part of routine clinical evaluation using a 3 T clinical scanner at 1 and 2 years of age. The imaging protocol included structural T1-weighted (T1-w) imaging, T2-weighted (T2-w) imaging, T2-FLAIR (FLuid Attenuated Inversion Recovery), and diffusion-weighted imaging (DWI) with and without gadolinium contrast enhancement. T1-w images were acquired using an Axial T1 protocol (voxel size: 0.47 × 0.47 × 5 mm^3^; TE/TR = 14/350 ms), providing high-resolution anatomical contrast for evaluating ventricular morphology and ChP architecture. T2-w images were obtained using a fast spin-echo (FSE) sequence (in-plane resolution 0.43 × 0.43 × 4 mm^3^, TE/TR = 106/2,717 ms), enabling assessment of cyst fluid content and perivascular structures. T2-FLAIR images were acquired using an inversion-recovery FSE sequence (voxel size: 0.47 × 0.47 × 5 mm^3^, TE/TR/TI = 8,000/125/2,000 ms), optimized to suppress the CSF signal and highlight periventricular white matter pathology. Diffusion-weighted imaging was performed using a single-shot echo-planar imaging sequence with two *b*-values (*b* = 0 and 1,000 s/mm^2^), 5 mm slice thickness, and in-plane resolution of 1.25 mm, slice thickness of 5 mm. Apparent diffusion coefficient (ADC) maps were generated to evaluate the diffusivity of the ChP cysts and surrounding tissues.

### 2.4 Postmortem MRI

The decedent underwent autopsy with an estimated postmortem interval (PMI) of less than 36 h. Following autopsy, the whole brain was removed and completely submerged in 10% neutral buffered formalin for fixation. The specimen was received in overall good condition; however, a portion of the left frontal lobe was unavailable due to prior sampling. The brain remained immersed in 10% formalin for approximately 5 months (164 days) prior to MRI acquisition.

A portion of the left frontal lobe was removed for separate neuropathological assessment, while the remaining left hemisphere and right hemisphere underwent imaging preparation. Prior to scanning, the hemisphere was rehydrated in phosphate-buffered saline (PBS) for approximately 2 weeks to wash out residual fixative and reduce fixation-induced tissue contrast alterations. After rehydration, the specimen was sealed in an airtight plastic bag filled with Fomblin (perfluoropolyether) to eliminate artifacts and prevent dehydration during MRI.

Whole-brain postmortem MRI was performed on a Siemens 3 T clinical scanner. The protocol included T1-weighted MPRAGE (Magnetization Prepared Rapid Gradient Echo, voxel size: 0.8 mm isotropic; TR/TE/TI = 2,100/2.14/500 ms), T2-w FSE (voxel size: 0.8 mm isotropic; TR/TE = 3,000/104 ms), T2-FLAIR (voxel size: 0.8 mm isotropic; TR/TE/TI = 2,100/2.14/500 ms), high-resolution gradient-echo (GRE) imaging (voxel size: 1.0–1.2 mm isotropic; TR/TE/TI approximately 4,000/392/1,100 ms), and multi-shell diffusion MRI (voxel size: 1.5 mm isotropic; TR/TE approximately 13,100/107 ms, 30 directions, *b*-values = 0, 3,000, 5,000, 7,000, 9,000 s/mm^2^) to evaluate structural integrity, ventricular anatomy, and cystic ChP morphology.

Following gross anatomical dissection, selected tissue blocks containing the ChP and surrounding structures were placed in standard plastic histopathology cassettes (dimensions 40 × 28 × 5 mm) and subsequently enclosed within an 80-ml syringe (Project, Wilson, NC, United States) to preserve hydration during high-resolution imaging over extended unattended scanning sessions. These tissue blocks were scanned on a 7 Tesla (7 T) Biospec 7030 preclinical MRI system equipped with a BGA-12S-HP shielded gradient coil insert (Bruker, Billerica, MA, United States) powered by high-performance gradient amplifiers (IECO, Helsinki, Finland) capable of generating 630 mT/m gradient strength with a 130-μs rise time. This setup enabled scanning using ultrahigh-resolution sequences, including T2-w FSE and T2-FLAIR imaging at 0.2-mm in-plane resolution, slice thickness of 1.5 mm. The 80-ml syringe was geometrically compatible with a Bruker mouse whole-body circularly polarized birdcage coil (model #T13161, inner diameter 40 mm) enabling uniform coverage with optimal performance for this setup. This histology-like MRI approach enabled microstructural visualization that closely approximates histopathological detail and facilitated improved localization for subsequent histological processing. However, contrast of postmortem MRI may differ from antemortem imaging due to postmortem effects, including fixation and PMI, which can alter MR relaxation properties and the diffusion microenvironment. Therefore, findings should be interpreted with caution.

### 2.5 Histopathological staining

Routine neuropathology examination was performed, three tissue blocks containing the ChP and white matter were processed, embedded in paraffin, and sectioned at 8 μm thickness. For routine histology, sections were stained with Luxol Fast Blue (LFB) or counterstained with hematoxylin and eosin (H&E) to assess overall tissue architecture, cyst morphology, calcification, and perivascular changes. LFB/H&E staining was performed by NYU Experimental Pathology. Immunohistochemistry for Aquaporin-1 (AQP1), cluster of differentiation 31 (CD31), collagen type IV alpha 1 chain (COL4A1), and smooth muscle actin (SMA) was performed by NYU Center for Biospecimen Research and Development (CBRD). Briefly, formalin-fixed, paraffin embedded (FFPE) tissue was sectioned at 8 μm. Sections were deparaffinized on the Ventana Medical Systems Discovery Ultra platform. AQP1 antigen retrieval was performed in Discovery Cell Conditioner 11 (Ventana Medical Systems #950–500) for 36 min at 95 °C. CD31 antigen retrieval was performed in Ultra Cell Conditioner 1 (Ventana Medical Systems #950-224) for 36 min at 95 °C. COL4A1 and SMA did not require antigen retrieval. Endogenous peroxidase activity was blocked with hydrogen peroxide for 4 min. Primary antibodies against AQP1 (Santa Cruz #sc-25287, 1:100) and COL4A1 (Millipore # AB769, 1:100) were diluted in antibody diluent (Ventana Medical Systems #ADB250) and incubated for 2 h at 37 °C. CD31 (Roche #760-4378) and SMA (Roche # 760-2833) were applied neat and incubated for 32 and 60 min, respectively. Antibodies were detected using Ultraview Universal conjugated goat anti-mouse/rabbit multimer-HRP or horse anti-goat multimer-horseradish peroxidase (HRP) followed by (3,3′-diaminobenzidine) DAB substrate (Ventana Medical Systems #760-500). All secondaries were visualized with ChromoMap RUO (760-159) DAB detection. Slides were washed in distilled water, counterstained with hematoxylin, dehydrated, and mounted with permanent media.

## 3 Results

### 3.1 Genetic evaluation using exome sequencing

The genetic analysis identified a heterozygous *de novo* missense variant in the *CERT1* gene (p. Ser260Leu; S260L). This variant was classified as pathogenic by the testing laboratory based on ACMG criteria. The p. S260L alteration is located in exon 5 of *CERT1.* This alteration results from a C to T substitution at nucleotide position 779, causing the serine (S) at amino acid position 260 to be replaced by a leucine (L). Functional studies have demonstrated abnormal CERT activity associated with this variant, consistent with a gain-of-function mechanism. No additional pathogenic or likely pathogenic variants relevant to the clinical phenotype were identified. No ACMG-reportable secondary findings were detected. In the genetic report, copy number variant analysis did not meet quality thresholds for gross deletion/duplication detection, and mitochondrial genome analysis was not included in this assay. The available evidence supports that the identified *CERT1* alteration is related to the patient’s clinical symptoms. However, no additional pathogenic variants explaining the sudden death were identified, and the precise cause of SUDC remains inconclusive. This *CERT1*-related special case complicated with SUDC may limit further pathological interpretation of the findings.

### 3.2 Clinical MRI findings

**Figure 1:**
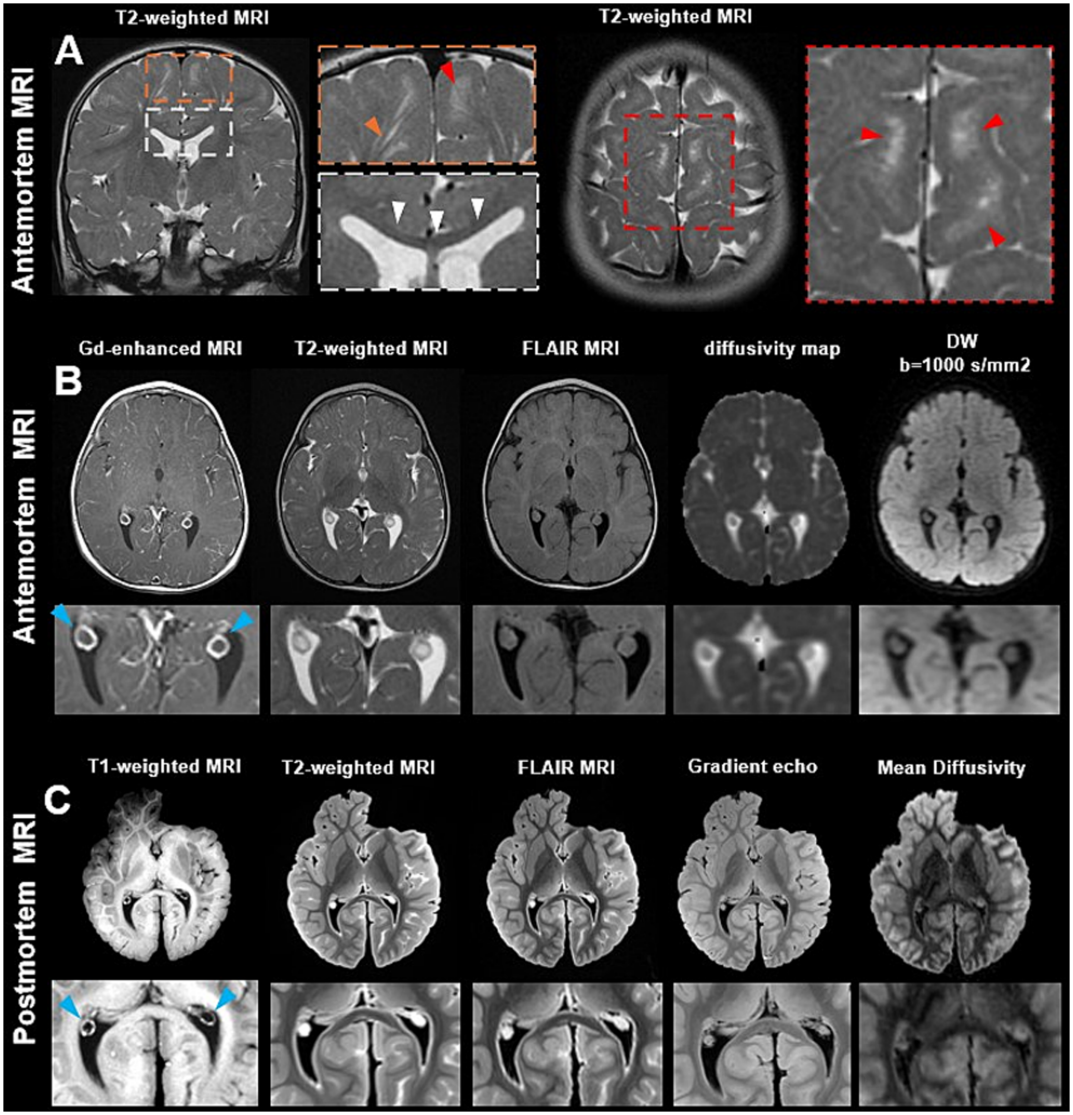
Antemortem clinical MRI demonstrates thinning of the corpus callosum and white matter hyperintensities, including enlarged perivascular spaces (orange arrowhead) and small areas of clustered tiny perivascular spaces (red arrowheads) on T2-weighted images **(A)**. Antemortem MRI **(B)** including gadolinium-enhanced T1-weighted, T2-weighted, and FLAIR images demonstrates symmetric cystic changes of the choroid plexus (ChP) with T2 hyperintensity and incomplete FLAIR suppression, indicating a non-CSF component. In addition, diffusivity/ADC maps and diffusion-weighted imaging (DWI, b = 1,000 s/mm^2^) show relatively low ADC values and increased DWI signal compared with CSF, consistent with restricted diffusion. Postmortem MRI **(C)** including T1-weighted, T2-weighted, FLAIR, gradient-echo, and mean diffusivity sequences, confirms the bilateral cystic morphology of the ChP. Notably, cyst size showed minimal change when comparing the antemortem MRI acquired at 2 years of age with the postmortem MRI obtained at 12 years of age.

Retrospective review of clinical brain MRI examinations obtained at 1 and 2 years of age revealed multiple structural abnormalities. On MRI obtained at 1-year-old, the brain demonstrates normal size and configuration for age, and the gray–white matter differentiation is preserved. The corpus callosum was diffusely thinned in particular noticeable in the genu on the coronal T2-weighted imaging (Figure 1A, white box), without focal agenesis or segmental dysgenesis. This finding was persistent and unchanged on one-year follow-up imaging, indicating a relatively stable developmental abnormality rather than a rapid progressive process. Mild underdevelopment of the inferior cerebellar vermis and subtle abnormalities of brainstem fiber tracts were also noted. The ventricular system and remaining midline structures were preserved. The basal ganglia, thalami, brainstem, and cerebellum are unremarkable. As shown in Figure 1A (orange and red boxes), the prominent PVS were identified with hyperintense T2 abnormalities diffusely distributed throughout both cerebral hemispheres, with a predilection for the juxtacortical and deep white matter regions. These PVS demonstrated irregular morphology and frequently appeared as a cluster of tiny tubular structures with coalescence into small, merged hyperintense foci. Notably, these signal abnormalities remained stable on follow-up imaging studies.

The ChP demonstrated bilateral and symmetric structural abnormalities characterized by heterogeneous signal intensity and cystic changes on MRI. The bilateral symmetry and diffuse involvement raised suspicion for an underlying systemic and intrinsic ChP structural alteration rather than an incidental or developmental cystic finding. As shown in Figure 1B, on post-contrast clinical T1-weighted imaging, both ChPs exhibited prominent ring enhancement with a relatively hypointense central component, consistent with cystic architecture as indicated on T2-weighted imaging. The enhancing peripheral rim likely reflects rich vascular ChP tissue surrounding centrally cystic regions. The zoomed-in images reveal a hypointense T1 and hyperintense T2 structure surrounded by ChP tissues. The iso-intense signal on FLAIR suggests that the contents of the cyst are not pure CSF, which would typically be suppressed and appear dark on FLAIR. This cyst-like structure appears slightly dark signal on the diffusivity map, indicating the restricted water diffusion as seen in other cysts that have high cellularity, keratinous/proteinaceous content, or viscous material. As a result of this cystic transformation, ChP appeared slightly bigger.

### 3.3 Postmortem MRI findings

High-resolution postmortem MRI of the whole hemisphere at 3 T preserved anatomical contrast comparable to antemortem clinical imaging. T1-, T2-, T2-FLAIR, and gradient-echo sequences (Figure 1C) demonstrated consistent tissue boundary delineation and signal characteristics, despite the fixation-related effects at the cerebral tissues, including increased T1 signal intensities, particularly within surface gray matter. In line with the *in vivo* findings, corresponding magnified postmortem images of the ChP revealed a cystic structure that showed no appreciable enlargement compared *in vivo* images acquired approximately 10 years earlier. It should be noted that mean diffusivity measurements between antemortem and postmortem MRI are not directly comparable due to changes in the physical microenvironment, including fixation-related tissue alterations and temperature differences.

**Figure 2:**
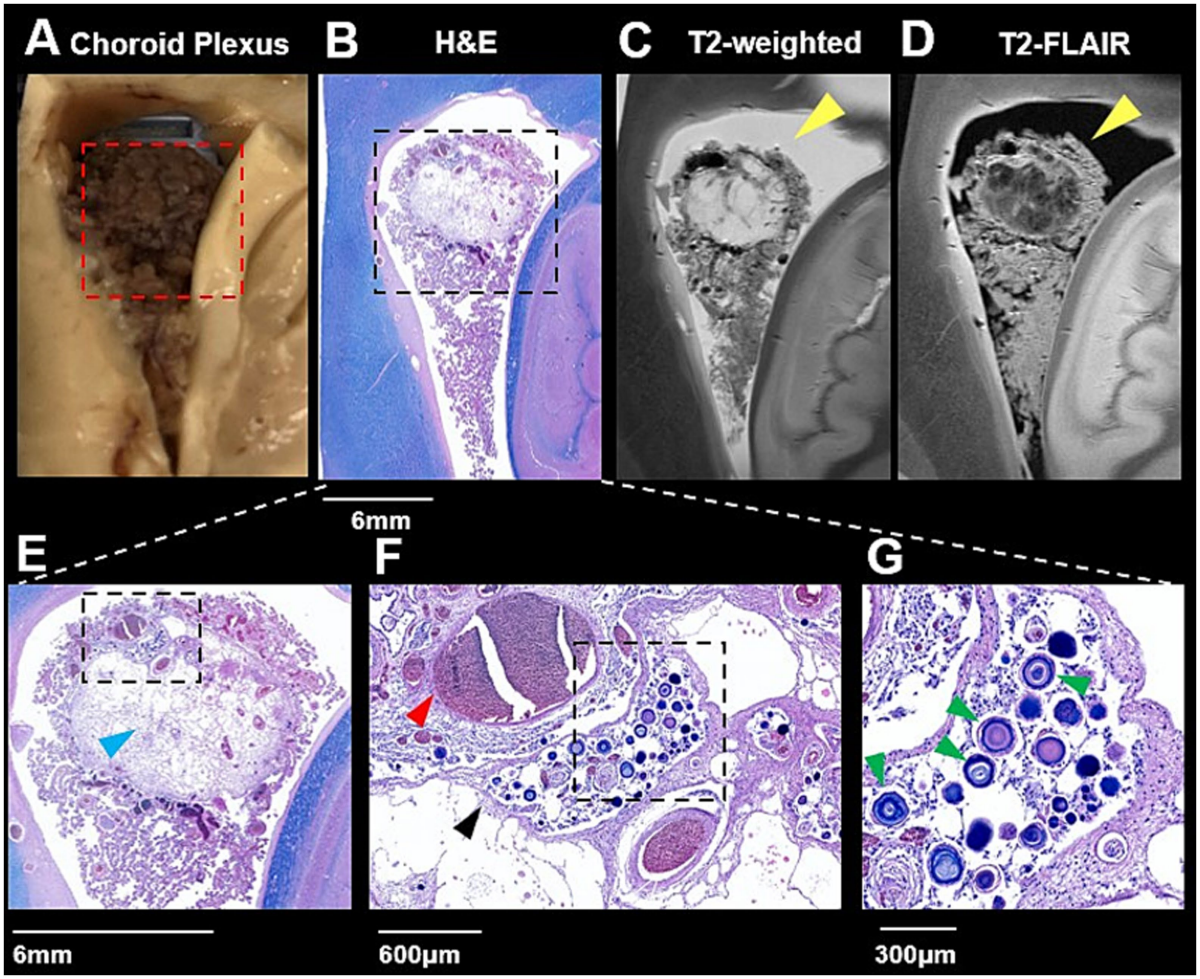
Histopathology-MRI correlation of choroid plexus (ChP) cysts. Gross examination **(A)** and LFB/H&E staining **(B)** revealed multiloculated ChP cysts, which correspond to hyperintense lesions on T2-weighted **(C)** and non-CSF component on FLAIR **(D)** MRI (yellow arrows). Higher-magnification H&E histology staining **(E–G)** highlights cystic spaces lined by surrounding disrupted epithelial cells (black arrowhead), displaced vessels (red arrowhead), pink proteinaceous material (blue arrowhead), and psammoma bodies or calcifications (green arrowhead).

Subsequent ultrahigh-resolution cassette imaging of dissected tissue specimens using a 7 T preclinical MRI system provided markedly enhanced microstructural details beyond what is achievable with clinical imaging. The 0.2-mm in-plane T2 and T2-FLAIR cassette images of the ChP enabled direct comparisons with histology, revealing fine anatomical features such as cyst walls, interstitial spaces, and blood vessels (Figures 2A–D). This ultra-high spatial resolution allowed precise alignment between MRI features and histopathological sections obtained from the center of the tissue block, supporting robust and reliable MRI–histology correlation of ChP pathology and the *in vivo* interpretations. The heterogeneous signal intensities observed on T2-weighted (Figure 2C) and FLAIR (Figure 2D) images correspond to detailed histopathological findings on H&E staining (Figure 2B). As shown in the magnified H&E images (Figures 2E–G), these include blood vessels of varying calibers (mostly capillaries) with intraluminal blood clots (Figure 2F), a thin-walled cyst containing fragmented vessels and eosinophilic, web-like material consistent with proteinaceous and viscous contents (Figure 2E), as well as psammoma bodies (concentric rings) or a cluster of calcifications of vessels walls adjacent to the cyst (Figure 2G).

### 3.4 Histopathological findings

**Figure 3:**
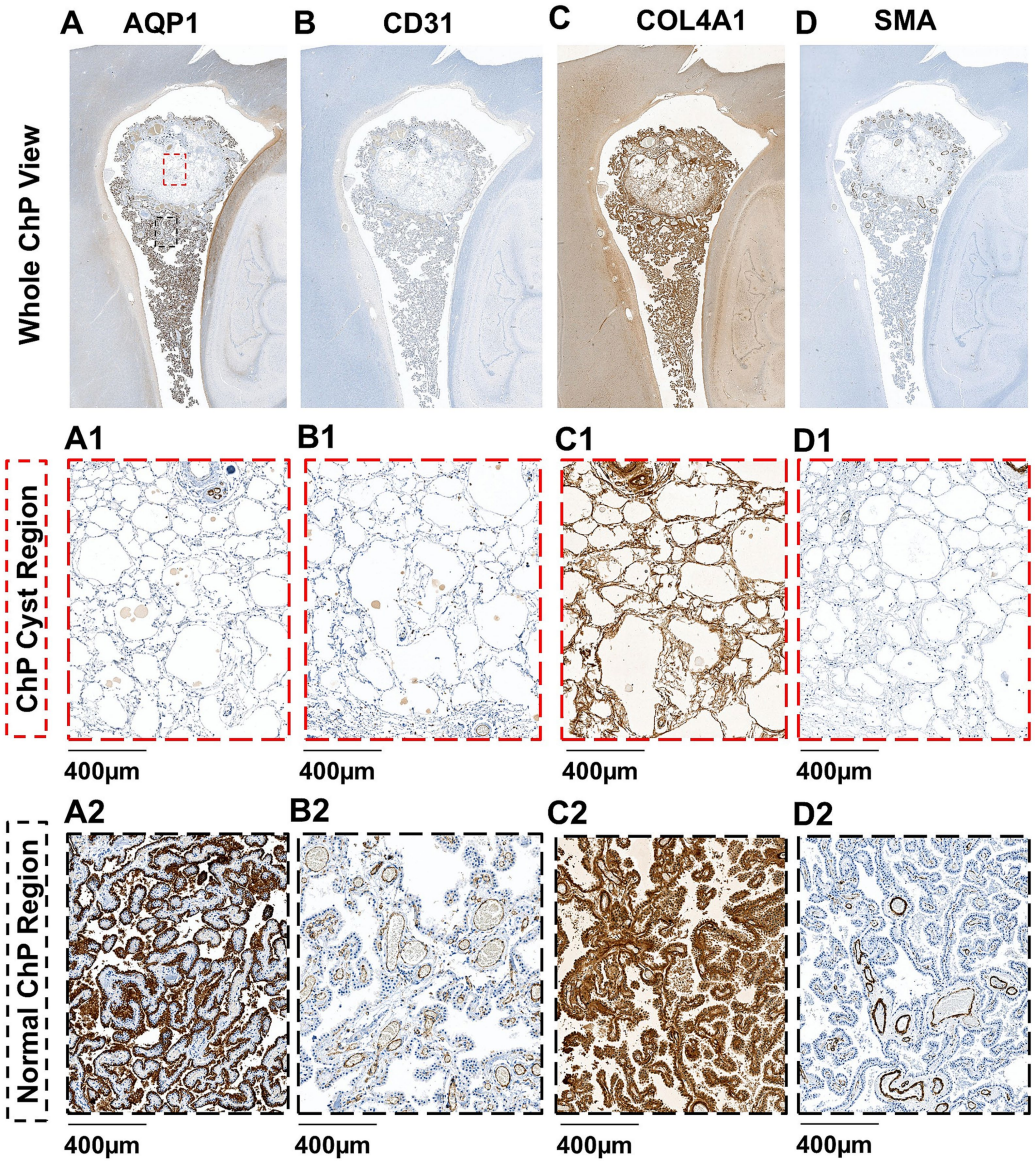
Immunohistochemical analyses of choroid plexus (ChP) cyst **(A–D)** include aquaporin-1 (AQP1) expression in ChP epithelium **(A)**, CD31 immunoreactivity highlighting endothelial cells **(B)**, collagen distribution revealed by COL4A1 staining **(C)**, and smooth muscle actin (SMA) expression delineating arterial smooth muscle **(D)**, as well as their corresponding higher-magnification views cystic region **(A1–D1)** and normal ChP region **(A2–D2)** highlighted within the red and red box in **(A)**, respectively.

Routine macroscopic and microscopic neuropathology examination revealed the presence of a ChP cyst, and widespread enlargement of PVS containing proteinaceous material throughout the subcortical white matter. Specifically, in addition to the LFB/H&E staining shown in Figure 2B, which demonstrated a cystic lesion with associated fibrotic changes, dense vessel wall calcifications, and disrupted cellular and vascular architecture in the surrounding tissue. Other immunohistochemical findings, including AQP1, CD31, COL4A1, and smooth muscle actin (SMA) staining (Figures 3A–D) provided further mechanistic insights into the observed abnormalities. AQP1 staining showed minimal positivity within the cystic region, suggesting potential impaired CSF-related water transport (Figure 3A). CD31 immunoreactivity revealed sparse and fragmented capillary networks within the cyst, indicative of compromised microvascular integrity (Figure 3B). COL4A1 staining demonstrated prominent collagen deposition, consistent with fibrotic remodeling (Figure 3C). Finally, SMA staining confirmed that larger arterial components were predominantly localized along the cyst periphery, highlighting a spatial reorganization of vascular elements associated with the cystic pathology (Figure 3D). These staining in the cyst region (Figures 3A1–D1) revealed distinct alterations compared with the normal ChP region (Figures 3A2–D2).

**Figure 4:**
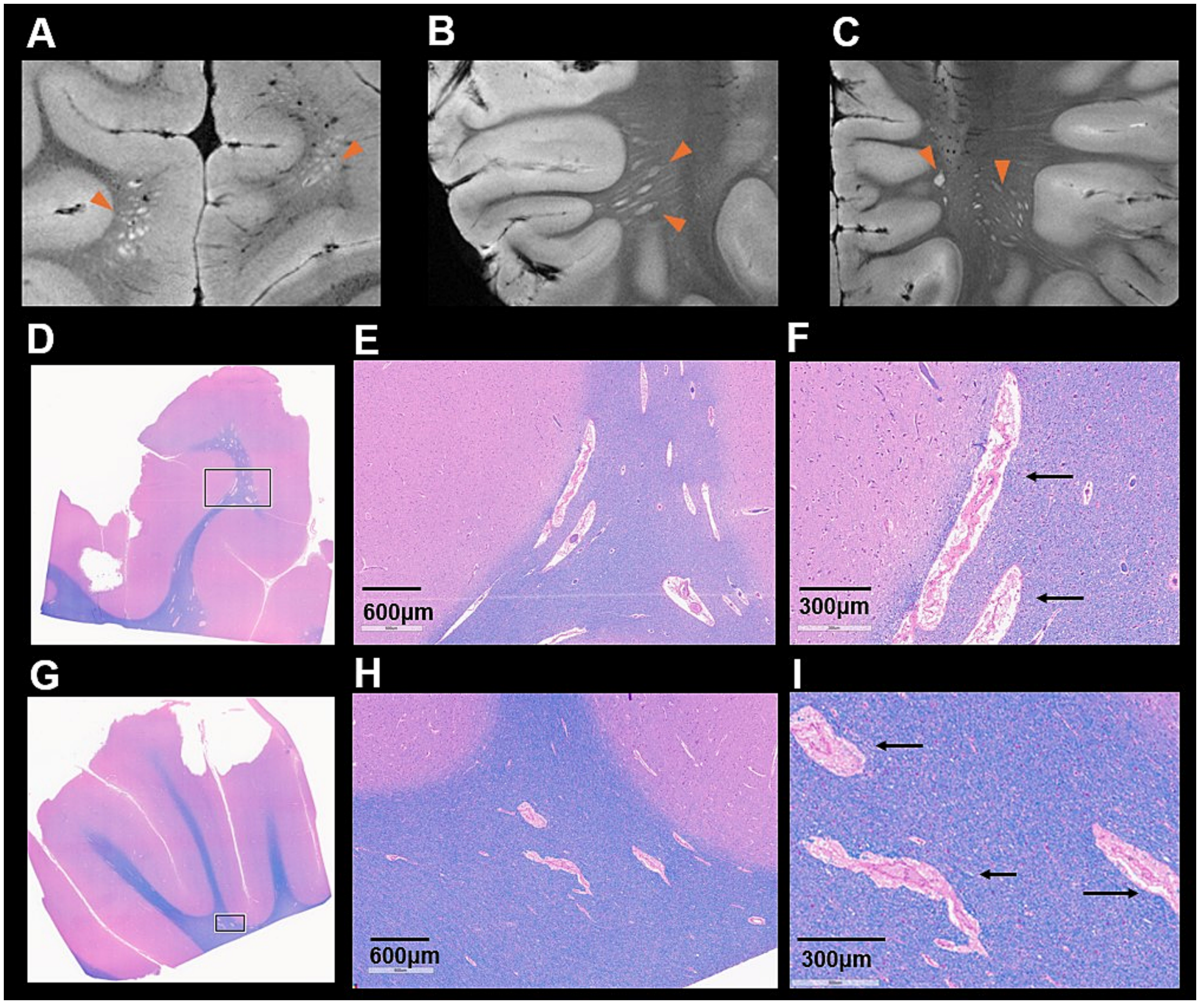
Postmortem high-resolution T2-weighted images **(A–C)** demonstrate prominent perivascular enlargement. LFB/H&E sections **(D,G)** show widespread perivascular abnormalities in white matter. Both low-magnification **(E,H)** and higher-magnification **(F,I)** views reveal enlarged perivascular space filled with pinkish proteinaceous material (arrows), indicating potential impaired perivascular clearance.

Additionally, the white matter and PVS abnormalities spatially appearing as hyperintense signals across multiple regions were also observed on high-resolution postmortem MRI (Figures 4A–C). In line with these findings, H&E staining of multiple subcortical white matter regions (Figures 4D–I) revealed clusters of abnormally dilated PVS of varying sizes containing eosinophilic, web-like material and disrupted vascular structures. Therefore, the findings revealed additional PVS abnormality along with ChP cysts.

## 4 Discussion

### 4.1 Abnormal ChP appearance

Consistent with prior study by Gehin et al. (3), neuroimaging in CerTra reveals a thin corpus callosum, ventriculomegaly, delayed myelination, and cerebellar atrophy. In our case, these expected features were observed, while additional findings, including ChP cysts and a pronounced PVS pattern, represent potentially novel imaging characteristics that have not been described in prior reports. Although abnormal sphingolipid metabolism is linked to myelin abnormalities, and lysosomal dysfunction, the potential involvement of *CERT1* variants in barrier or membrane functions related to CSF dynamics has not been previously described. Given that CerTra syndrome arises from *CERT1* variant and dysregulated sphingolipid homeostasis, a process fundamental to membrane composition, endothelial cell integrity, and neurovascular signaling, these molecular disturbances may extend to the integrity and function of fluid-regulating structures (6, 20), including the blood–brain barrier (BBB) and blood–CSF barrier (BCSFB). Both barriers rely on highly ordered lipid architecture and specialized endothelial–epithelial interactions. For instance, in certain lipid-storage disorders such as Niemann–Pick disease type C (NPC), which is characterized by defective intracellular lipid and cholesterol trafficking, the ChP has been shown to be directly affected. In NPC animal models, ChP epithelial cells exhibit marked accumulation of intracellular vesicles and autophagosomes, along with altered release of extracellular vesicles into the CSF, leading to deleterious downstream effects on brain parenchyma (21). These observations indicate that disruptions in lipid trafficking can impair ChP structure and function, disturbing CSF homeostasis. In *CERT1*-related abnormalities, several studies (6, 7) have shown that sphingolipids are crucial for endothelium formation, a critical component of the blood–brain-barrier. These abnormalities may involve the ChP, where membrane continuity, vascular compliance, and ionic regulation of endothelial cells of ChP are essential for normal physiology and CSF homeostasis.

In our case with a *CERT1* variant, T2-weighted MRI demonstrated ChP cysts (Figures 1B,C). Although ChP cysts are often regarded as benign and clinically insignificant, emerging evidence suggests that cystic changes may be more prevalent in individuals with mild cognitive impairment and neurodegenerative disorders such as Alzheimer’s disease (AD) (16–18, 22). These cysts may reflect disrupted CSF production or altered fluid dynamics, potentially contributing to impaired glymphatic waste clearance. Histopathological evaluation of the cystic region further revealed fibrotic architecture and clustered calcifications on H&E staining (Figure 2B). Immunohistochemical markers related to ChP and BCSFB function, including CD31 for capillary endothelium, SMA for larger vessels, and AQP1 for CSF-secreting epithelium, showed markedly reduced or absent expression within the cystic area, indicating local loss of BCSFB integrity, which further provides pathological evidence to show potential BCSFB functional impairment in this case (Figures 3A–D).

### 4.2 Abnormal PVS appearance

Furthermore, PVS appeared as another site that exhibits abnormality on MRI and histopathology. As an important glymphatic pathway, small vessels within the PVS may undergo vascular alterations driven by sphingolipid dysregulation. Sphingolipid imbalance has been shown to disrupt endothelial tight junctions of BBB and compromise vessel wall, leading to increased vascular leakage. As shown in both antemortem and postmortem MRI revealed abnormal PVS appearance on T2-weighted images, along with diffuse abnormal white matter signals. LFB staining further revealed increased number of enlarged PVS, protein-rich, debris-filled PVS in this patient (Figures 4D–I), which may represent a downstream consequence of disrupted membrane of small vessel and altered CSF flow homeostasis in *CERT1*-related disease. These changes may lead to stagnation of interstitial fluids within the PVS due to accumulation of protein-rich debris, or associated edema, thereby changing the CSF flow dynamics or pressure. These findings were observed in the context of a pathogenic *CERT1* gain-of-function variant and may reflect alterations in perivascular structure. As phenotypical changes, these processes could manifest radiologically as enlarged PVS or impaired perivascular fluid dynamics, reflecting subtle but significant small-vessel pathology (23). Although speculative, these abnormalities are biologically plausible given the central role of sphingolipids in maintaining stability of BBB integrity and fluid-dynamic homeostasis, and they may represent structural correlations of barrier dysfunction in CerTra syndrome.

### 4.3 Limitations

This study has several important limitations. First, as a single-case report, the findings are descriptive and correlative, and causality between the identified *CERT1* variant and the observed ChP and PVS abnormalities cannot be fully established. Additional confounding factors related to the unexplained sudden death and patient’s medical history may have complicated the neuropathological findings. Additionally, although a cardiac assessment was performed during the autopsy, toxicology testing and detailed functional cardiac evaluation were not comprehensive. Therefore, the ability to further evaluate potential contributing factors remains limited. However, in cases complicated by sudden unexplained death, determining the exact cause of death remains inherently challenging due to the absence of definitive pathological or clinical findings. Moreover, given the rarity of *CERT1*-associated CerTra syndrome and SUDC, the generalizability of these observations remains uncertain and challenging to be evaluated. The interpretation of the observed histological features remains speculative, as these findings may not be specific to *CERT1* variants and may also be seen in other conditions, including chronic hypoxia, age-related or metabolic changes, and storage disorders. Further mechanistic studies are needed to establish causality. Second, neuropathological interpretation may be influenced by postmortem effects, such as PMI, tissue handling, and fixation-related changes. These factors can alter tissue morphology and potentially affect assessment of ChP structure and PVS. Although standard procedures were followed, postmortem effects cannot be fully excluded (24). Third, altered AQP1 expression should be interpreted with caution, as decreased AQP1 may be not disease-specific. Experimental studies have demonstrated that changes in intracranial pressure states, including hydrocephalus, can modulate AQP1 expression in ChP epithelium (25). Therefore, altered CSF dynamics or pressure-related factors may represent potential confounders independent of the underlying genetic variant. While no definitive evidence of hydrocephalus was identified in this case, pressure-related mechanisms cannot be entirely excluded. Finally, the rarity of CerTra syndrome makes it harder for a large-scale study. Future studies involving additional cases and alternative experimental approaches will be necessary to better characterize the neuropathological spectrum associated with *CERT1* variant.

## 5 Conclusion

In summary, ChP cystic changes and PVS abnormalities, beyond known MRI features (such as corpus callosum thinning and ventriculomegaly) in *CERT1* variant, represent a previously unreported neuroimaging phenotype. *CERT1* variants may lead to sphingolipid imbalance, which is hypothesized to cause ChP/BCSFB dysfunction and PVS/glymphatic alterations, ultimately impairing brain homeostasis and increasing neurological risk.

## Data Availability

The original contributions presented in the study are included in the article, further inquiries can be directed to the corresponding author.
